# Long-Term Mesophilic Anaerobic Co-Digestion of Swine Manure with Corn Stover and Microbial Community Analysis

**DOI:** 10.3390/microorganisms8020188

**Published:** 2020-01-29

**Authors:** Haipeng Wang, Teng Teeh Lim, Cuong Duong, Wei Zhang, Congfeng Xu, Lei Yan, Zili Mei, Weidong Wang

**Affiliations:** 1 Heilongjiang Provincial Key Laboratory of Environmental Microbiology and Recycling of Agro-Waste in Cold Region, College of Life Science and Biotechnology, Heilongjiang Bayi Agricultural University, Daqing 163319, China; wanghaipenglab@163.com (H.W.); zhangwei19900131@126.com (W.Z.); 13674591462@163.com (C.X.); hekouyanlei@gmail.com (L.Y.); 2 Agriculture Systems Management, Division of Food Systems and Bioengineering, University of Missouri, Columbia, MO 65211-5200, USA; limt@missouri.edu (T.T.L.); cmdyy6@mail.missouri.edu (C.D.); 3 Key Laboratory of Development and Application of Rural Renewable Energy, Ministry of Agriculture and Rural Affairs, Chengdu 610041, China; m13618016610@163.com

**Keywords:** biogas, semi-continuous digesters, swine manure, farm waste, Amplicon sequencing

## Abstract

Long-term anaerobic co-digestion of swine manure (SM) and corn stover (CS) was conducted using semi-continuously loaded digesters under mesophilic conditions. A preliminary test was first conducted to test the effects of loading rates, and results indicated the 3 g-VS L^−1^ d^−1^ was the optimal loading rate. Based on the preliminary results, a verification replicated test was conducted with 3 g-VS L^−1^ d^−1^ loading rate and different SM/CS ratios (1:1, 2:1 and 1:2). Results showed that a SM/CS ratio of 2/1 was optimal, based on maximum observed methane-VS_des_ generation and carbon conversion efficiency (72.56 ± 3.40 mL g^−1^ and 40.59%, respectively). Amplicon sequencing analysis suggested that microbial diversity was increased with CS loading. Amino-acid-degrading bacteria were abundant in the treatment groups. Archaea *Methanoculleus* could enhance biogas and methane productions.

## 1. Introduction

There is an abundant supply of swine manure (SM) on livestock farms that contains plentiful organic matter, which may be used as feedstock in anaerobic digesters. However, the carbon/nitrogen (C/N) ratio for SM is around 6 to 8, depending upon pig growth stage, which is often too low for an anaerobic digestion (AD) to efficiently utilize the nutrients and function properly [1]. In addition, manure solid-liquid separation is becoming popular due to a combination of cost reduction and high removal efficiency [2]. AD is a process that converts organic matter (e.g., animal manure, agricultural residue, municipal waste, etc.) into a gaseous mixture, mainly composed of methane and carbon dioxide, through the concerted action of a close-knit community of bacteria [3]. Nevertheless, AD of single substrates (mono-digestion) presents some drawbacks linked to substrate properties. For instance, animal manure tends to have low organic loads and high nitrogen (N) concentrations, which may inhibit methanogens, while agro-industrial wastes are often seasonal substrates, which might lack nitrogen. Co-digesting corn stalk with pig manure showed higher methane production than mono-digestion [4], and it was found that manure could provide buffering capacity and a wide range of nutrients. Much of these inefficient digestion problems can be improved by the addition of a co-substrate in what has been recently come to be called anaerobic co-digestion [5].

Corn is one of the most important cereals in the world, accounting for an average production of 840 million metric tons year^−1^ in the last decade [6]. Considering an average harvest index of 50%, a similar proportion of stover productivity can potentially be achieved thus making corn the most abundant source of cereal stover [7]. In addition, corn stover (CS) consists mainly of cellulose, hemicellulose, lignin, ash and extractives and high carbon [8]. Some countries are encouraging better utilization of the CS, because some of the small producers tend to burn the CS in the field to prepare for the next planting. The burning can cause air pollution issues while reducing soil organic matter. Generally, SM contains a high total nitrogen (TN), which decreases the C/N ratios when anaerobic co-digestion with CS, which is beneficial to the anaerobic co-digestion.

Co-digestion of SM and CS was first studied by Fujita et al., the addition of CS to SM enhanced biogas production in both mesophilic (39 °C) and thermophilic (55 °C) anaerobic digestion [9]. More than 50% of the carbon in CS was converted to gas. Wu et al. studied three crop residues (oat straw, wheat straw, and corn stalks) in anaerobic co-digestion with SM, among which corn stalks performed the best with an increase in daily maximum biogas volume by 11.4-fold compared to the control (SM only) at mesophilic condition [10]. Cuetos et al. [11] demonstrated SM and maize gave the highest CH_4_ yields in batch digestion tests in comparison with rapeseed and sunflower. Zhang et al. [12] studied archaeal microbial dynamics of anaerobic co-digestion cattle manure liquid with corn straw and found corn straw was mainly metabolized by acetate-utilizing methanogens, with *Methanosaeta* as the dominant archaeal community. Gao et al. [13] also found *Methanosaeta* was the dominant methanogen in a dairy manure production-scale biogas plant. Literature that documents long-term semi-continuous and microbial community of anaerobic co-digestion of SM and CS is limited outside of the research cited, suggesting that additional exploration into the function and composition of co-digestion communities is warranted. 

The objectives of this paper were to systematically monitor the long-term and stable biogas production of co-digesting SM with CS, in semi-continuously loaded lab-scale digester. By adjusting manure to stover ratio in fixed VS loading (2, 3 and 4 g-VS L^−1^ d^−1^), and characterizing biogas and methane productions, and microbial community in the different ratios of SM/CS digesters, pH value and other variables were also recorded. This will be helpful for the practical industrial application of anaerobic co-digestion.

## 2. Materials and Methods 

The experiments were conducted at the University of Missouri College of Agriculture, Food and Natural Resources (Columbia, MO, USA). The digesters consisted of wide-mouth 1.89 L (0.5 gallon) glass bottles with a working volume of 1.38 L. Each digester was equipped with a rigid plastic duct for pumping nitrogen and collecting biogas, which was connected with soft plastic pipe. The digesters were maintained at 38 ± 1 °C. A 21 days’ hydraulic retention time (HRT) was achieved by adding/removing the same volume of substrate/digestate (131 mL) every 2 days. Biogas volume and CO_2_ concentration were measured every 4 days. The pH value was measured every 2 days. The initial inoculum volume was half the working volume (0.89 L). All jars were swirled three times (8:00 am, 12:00 pm and 6:00 pm) per day to enhance mixing.

### 2.1. Feedstock Materials

Fresh SM in this study was collected from a grow-finish barn in central Missouri (USA). The farm raised antibiotic-free pork, and had a solid-liquid separation manure collection system that separated a significant portion of solid manure. The manure samples used in the tests were collected from the solid manure storage pile. The collected manure was stored in 5 gallon (18.9 L) buckets, with air-tight lids, and kept frozen at −20 °C. The frozen manure was thawed for about 24 h at room temperature prior to use. A sample from each bucket was collected and measured for total solids (TS) and volatiles solids (VS) contents, following EPA method 1684 [14]. The TS of SM was 25.89%, and the VS was 82.02% of the TS.

The CS sample was obtained from a corn field of South Farm Research Center (Columbia, MO, USA) in November, 2016. The CS sample was first cut into 6–8′′ (20–27 cm) sections, and were then ground to the size of 8-mesh (2.36 mm) by an electric grinder. The ground CS was stored in dry black plastic bags and at room temperature until use. Samples of the ground CS were sent to the University of Missouri Agricultural Experiment Station Chemical Laboratories for cellulose and lignin content analysis. Measurements of the cellulose and lignin contents were conducted using published standard methods [15].

The inoculum was collected from semi-continuous anaerobic digestion jars that had been steadily producing biogas for over three months with an organic loading rate (OLR) of 2 g-VS L^−1^ d^−1^. The average biogas production was about 1246 ± 515 mL d^−1^. The inoculum had a TS of 2.20%, and a VS that was 64.92% of TS.

### 2.2. Starting Up of the Digesters (Test 1)

A preliminary test (Test 1) was first conducted to compare the effects of three loading rates (2, 3 and 4 g-VS L^−1^ d^−1^) and three SM/CS ratios (SM/CS = 1:1, 2:1, and 1:2) onto biogas and methane production. A total of 12 digesters were used in Test 1, which were three control digesters, and three groups of digesters for three VS loading rates, and within each group there was one each with the three SM/CS feedstock ratio. Naming of the digesters was based on the VS loading rate, followed by the SM/CS ratio. For example, a digester that was loaded with 3 g-VS L^−1^ d^−1^ and SM/CS ratio of 1:2 was labeled as digester 3-1-2. 

The preliminary test digesters with 2 g-VS L^−1^ d^−1^, but the digesters with higher loadings (3 and 4 g-VS L^−1^ d^−1^) were immediately loaded with the corresponding feedstock, without a stabilizing or adopting period. Most of the digesters (except for the three controls and 2-1-1, 2-1-2 and 3-1-2), appeared to have stopped producing biogas when the pH values dropped below 6, which occurred within the first 10 days. The failed digesters were restarted using the digestate of working digester, and allowed to undergo a longer start-up and stabilization period. 

**Figure 1:**
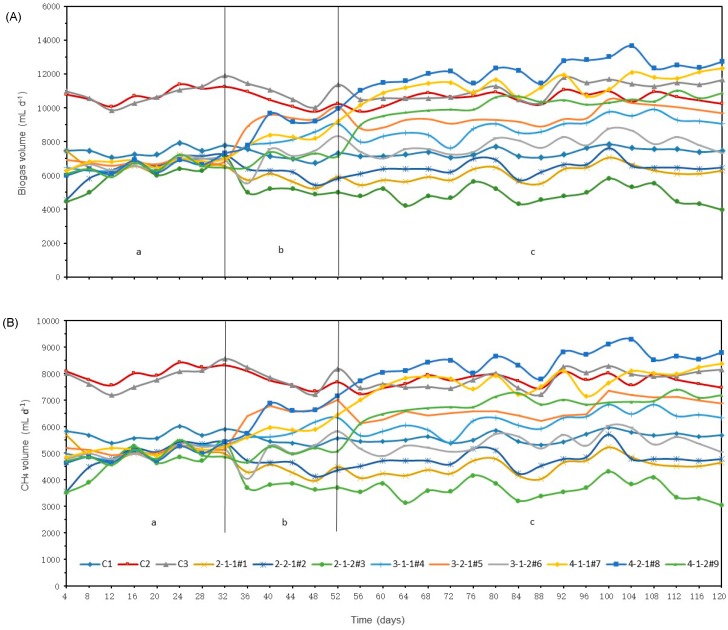
Biogas (**A**) methane (**B**) production rates of the control (C1-C3) and treatment digesters, periods a and b were when the digesters were loaded with 2 and 3 g-VS L^−1^ d^−1^, and period c was when all digesters were loaded following the experiment design.

Test 1 was conducted for 5 HRT (105 days). Each jar was flushed with nitrogen for 30 s at a flow rate of 20 L/min before being sealed and placed into an incubator after initial feedstock addition. At the first 32 days (Figure 1, period a), all treatment digesters were loaded with 2 g-VS L^−1^ d^−1^, After that, #4 to #9 (Figure 1, period b) were loaded following the loading rate of 3 g-VS L^−1^ d^−1^ for 1 HRT (21 days). Digesters #7, #8, and #9 (Figure 1, period c) were loaded with the 4 g-VS L^−1^ d^−1^ of feedstock, VS feedstock was 84 g L^−1^, meaning all digesters were loaded with the designated VS loading rates and SM/CS ratios. Digester jar #1 was 2-1-1 (that VS loading was 2 g-VS L^−1^ d^−1^ while SM/CS was 1:1), jar #2 was 2-2-1, and jar #3 was 2-1-2, and so on.

### 2.3. Verification Experiments (Test 2)

Based on the results of Test 1, a complementary test (Test 2) was conducted to provide higher statistical confidence power of tests among the co-digestion treatments and a promising VS loading rate of 3 g-VS L^−1^ d^−1^, based on observed biogas production per VS-destroyed and methane production. Test 2 was conducted for 4 HRT (84 days), with three SM/CS ratios (SM/CS = 1:1, 2:1, and 1:2) and controls (VS loading = 3 g-VS L^−1^ d^−1^, SM only, *n* = 3), for a total of 12 jars. Other conditions were the same as in Test 1.

### 2.4. Digestate and Biogas Analysis

Biogas produced from each digesters was collected using 10-L Tedlar bags, which was measured for volume every 4 days. The volume of biogas was estimated directly by measuring height of bags and applying a bag height-to-volume relationship equation. Said equation was established by relating the height of three bags filled with various volumes of nitrogen gas and room air to their respective volumes using a precision volume syringe [16].

Total solids and VS of the feedstock and digestate were analyzed following EPA method 1684 [14]. Methane concentration was measured indirectly by subtracting carbon dioxide (CO_2_) concentration from 100%. The CO_2_ concentration was measured by using a CO_2_ analyzer (FYRITE, Bacharach, New Kensington, PA, USA). The pH of digestate was measured every 2 days, with a pH monitor (PINPOINT, American Marine Inc, Ridgefield, CT, USA). Calibration was conducted by using two known pH solutions (4.0 and 7.0). Before measuring pH, each digester was swirled thoroughly, and the pH probe was kept in the digestate for over 1 min to ensure equilibrium before readings were taken. Total alkalinity (TA) measurement was conducted by using an Alkalinity Test Kit (HACH, AL-AP MG-L, Loveland, CO, USA). Digestate sample was diluted at 1:100 ratio by using distilled water. Ammonia concentration was measured using ammonia (un-ionized ammonia (NH_3_) and the ammonium ion (NH_4_^+^) Test Kit (HACH, NI-SA), and the digestate was typically diluted at 1:3000 level. The result was reported in mg L^−1^ as calcium carbonate.

### 2.5. Microbial Community Analysis

For microbial community analysis, fresh digestate (containing liquid and solid portions) samples were collected from each of the 12 jars in Test 2 and sent to University of Missouri Metagenomics Laboratory. Firstly, samples were mechanical disrupted using a tissue lyser, centrifuged at 5000× *g* at room temperature, added with ammonium acetate, incubated on ice, and centrifuged again. Secondly, supernatant was mixed with one volume of chilled isopropanol and incubated for 30 min on ice. Finally, the analysis will follow standard operating procedures for the mechanical disruption, incubation, centrifugation, DNA sample purification and identification, and data analysis [17].

Extracted digestate DNA was processed at the University of Missouri DNA Core Facility. Bacterial 16S rRNA was amplified using modified universal primers 515F (5′-GTGYCAGC MGCCGCGGTAA-3′) [18] and 806R (5′-RGGACTACNVGGGTWTCTAA-3′) [19]. Barcoded sequence data was filtered, trimmed, and annotated against a database of bacterial 16S at the MU Informatics Research Core Facility. Contiguous sequences of DNA were assembled using FLASH software.

QIIME version 1.9.1 software was used to perform de novo and reference-based chimera detection and removal, and remaining contigs were assigned to Operational Taxonomic Units (OTUs) using a 0.03 OTU definition (97% sequence similarity cut-off level). Taxonomy was assigned to selected OTUs using BLAST against the Greengenes database of 16S rRNA gene sequences.

### 2.6. Data Preparation and Statistical Analysis

Spreadsheet software (Excel 2010 and Origin 8, Microsoft Corporation, Redmond, WA, USA) were used for analyzing and graphing. A statistics software package, SPSS Atatistics 22 (SPSS Inc., IBM, Armonk, NY, USA) was used for analyzing correlation of different items. Analysis of variance (ANOVA) was used for analyzing significant difference among treatments. 

## 3. Results

### 3.1. Test 1―Starting up of the Digesters

#### 3.1.1. Biogas and Methane Production

Biogas production rates of every four-day period are presented, and divided into periods a, b, and c for comparisons (Figure 1A). During period a, all VS loading rate (except of control #2, C2, and control #3, C3) was kept the same as the control digester of 2 g-VS L^−1^ d^−1^, the biogas productions were 1500–1750 mL d^−1^, which compared fairly well with the control digester at 1,654.5 mL d^−1^. In period b, the VS loading rate of the treatment digesters was increased to 3 g-VS L^−1^ d^−1^. After only 8 days, the biogas volume already showed an increasing trend following the increased VS loading rate, when compared with the control digesters. After loading as designed in period c, the biogas production rates of jars with 4 g-VS L^−1^ d^−1^ (#7, #8 and #9) rapidly increased to higher values with respect to the control digesters, and stabilized in less than 1 HRT (21 days).

Results presented in this paper are based on period c post-equilibrium (72–120 days), Figure 1A. Biogas production rates of #7 and #8 were higher than C3, by 2.6% and 9.9%, respectively. The average biogas productions of C1, C2 and C3) were 1856, 2658 and 2804 mL d^−1^, respectively. Biogas production of C2 was 30.2% higher than C1, while C3 was only 5.2% higher than C2, which indicates that the biogas production was not proportional to the VS loading rate of SM. Furthermore, the biogas production per VS-destroyed (VS _des_) can reflect the efficiency of digester in utilizing the feedstock. When comparing the biogas-VS _des_ among the controls, they were 97.8, 112.0, and 92.2 mL g^−1^-VS _des_, for C1, C2, and C3 respectively. Although C3 produced the most biogas volume, it had the lowest utilization of feedstock. The results suggest that the biogas production efficiency is best by considering both VS loading and system limitation. The relatively lower biogas production rate at the 4 g-VS L^−1^ d^−1^ loading rate was most likely due to the digester retention time, volume and quantity of microorganisms.

Comparing the different SM/CS ratios, the SM/CS of 2/1 produced more biogas than other ratios per VS loading rate, in general, biogas production rate was SM/CS = 2/1 > SM/CS = 1/1 > SM/CS = 1/2 (Table 1). This indicates that the CS contained complex organic matter, i.e., cellulose, hemicellulose and lignin [20] and most of the organic matters were more difficult to digest in the AD process. The most biogas volume was measured for the 4-2-1#8 setting on 104 days, which was 3415 mL d^−1^. The minimum biogas volume was recorded for the 2-1-2#3 treatment on 84 days, which was 1083 mL d^−1^. Comparing all the treatments, the 2-1-1#1 digester had the highest biogas per VS destroyed, at 115 mL g^−1^-VS _des_, although due to the limited nutrients (VS) in digester #1, it did not produce the most biogas. The biogas-VS _des_ of digester 4-2-1#8 was 101 mL g^−1^-VS _des_, the VS loading was 4g L^−1^ d^−1^, which produced the most overall biogas, although the digestion efficiency was not proportional to the VS loading rate. As a comparison, the C1 digester was added with 26.8 g of SM every 2 days, while the digester 4-1-1#7 was added with 26.8 g of SM and 6.4 g of CS. Both digesters had same quantity of SM added, but with different amounts of CS and dilution water. Nevertheless, the digester 4-1-1-#7 produced 2,878 mL d^−1^ of biogas, which was only 35.5% higher than the C1 digester (4 vs. 2 g-VS L^−1^ d^−1^), at 1,856 mL d^−1^ of biogas. The digester 4-1-1-#7 produced 1,952 mL d^−1^ of methane, and the C1 digester produced 1405 mL d^−1^ of biogas, or 28.0% more than C1. These findings confirmed the feasibility to co-digest SM with CS, although one should expect lower digestion efficiency for the CS feedstock.

The methane generation of C1, C2 and C3 was observed to have decreased almost proportionally (Table 2) such that 1g VS loading difference equated to an approximate was about 2.5% methane content less when comparing C1 with C2, and a 2.63% decreased comparing C2 with C3. For the treatment digesters, methane concentrations for the 2 and 3 g-VS L^−1^ d^−1^ were similar, averaging 74% and 70%, respectively. For treatment digesters with VS loading of 4 g-VS L^−1^ d^−1^, the methane concentration of jar 4-2-1#8 was 69.13% higher than 4-1-1#7 (67.88%) and 4-^1^-2#9 (67.25%). The highest methane volume was produced by the digester of 4-2-1#8 (104 days), which was 2,147 mL d^−1^. The lowest biogas volume was produced by the 2-1-2#3 (84 days) at 909 mL d^−1^. On average, digester 4-2-1#8 produced the most methane (2,147 mL d^−1^), but the digester 2-1-1#1 produced the highest methane volume per VS _des_ (84.4 mL d^−1^), most likely limited by the lower feedstock nutrients.

#### 3.1.2. Variation of pH, total alkalinity and ammonia

The pH values of digesters C2 and C3 were similar, and averaged 7.87 and 7.86, respectively, just slightly higher than C1, of 7.61 (Table 2). For all treatment digesters, digester 4-2-1#8 had the highest pH value, averaged 7.72, while digester 2-1-2#3 had the lowest pH, which was 7.08. In general, pH tended to be higher for the those that contained more SM, including those of the 3 g-VS L^−1^ d^−1^ and 4 g-VS L^−1^ d^−1^. The higher pH values were caused by the high organic matters with SM, which was also producing higher biogas generation.

Total alkalinity of the controls was higher than all the treatments, regardless of VS loading (Table 2). The higher SM content contributed to higher TA, C3 had the highest TA, at 18,000 mg CaCO_3_ L^−1^. Ammonia concentrations of the control digesters were also higher than treatment digesters. The ammonia content of all treatments was similar, 800 mg NH_3_ L^−1^, except 4-2-1#8 was 1600 mg NH_3_ L^−1^. Ammonia concentration of C3 was 4000 mg NH_3_ L^−1^.

### 3.2. Test 2―Verification experiments

#### 3.2.1. Biogas and methane production

**Figure 2:**
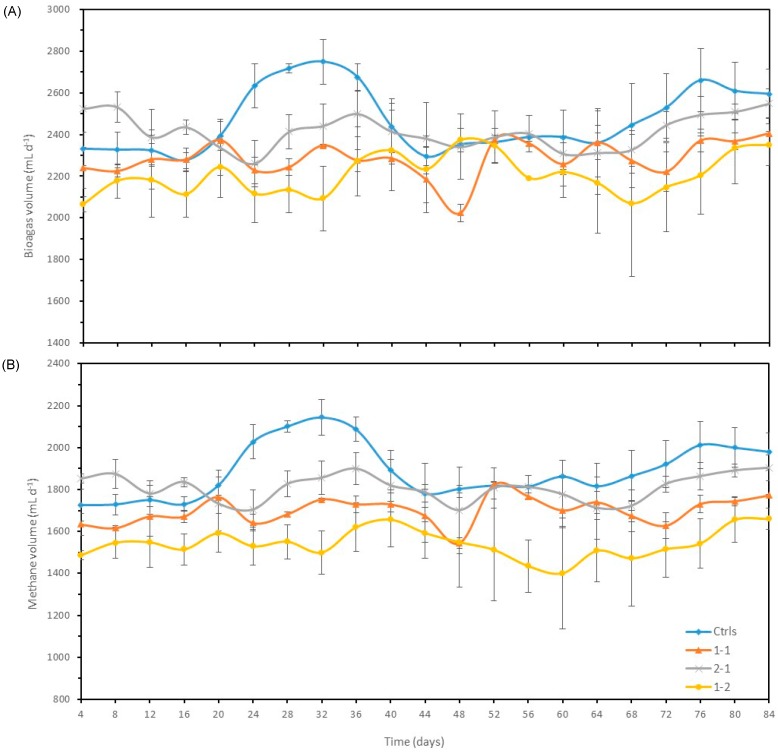
Variation of biogas (A) and methane (B) production of control and treatments in Test 2.

Variation of biogas and methane productions at a same VS loading rate (3 g-VS L^−1^ d^−1^) but with different ratios of SM/CS (1:1, 2:1 and 1:2) were averaged and are presented in Figure 2A,B, respectively. During the early period of this AD test, the control digesters were lagging behind the 2-1 ratio, but peaked for about three weeks. This indicates that the microorganisms of the control digesters needed more time to adapt to a new loading rate, and the co-digestion of SM and CS showed better adaptability than the controls, and better carbon and nitrogen balances.

After that, the control digesters produced higher biogas than the others in second HRT and the trend was similar to those of Test 1. In general, greater CS in the feedstock result in lower the biogas and methane contents, especially towards the end of the monitoring period. The average biogas production of Controls (2415 mL d^−1^) was identical to 2-1 (2,415 mL d^−1^), but significantly higher (*p* < 0.01) than 1-1 (2284 mL d^−1^) and 1-2 (2166 mL d^−1^), respectively. 

In Table 3, the average methane percentages of Control, 2-1, 1-1, and 1-2 showed a downward trend (76.6%, 75.0%, 74.4%, and 71.3%, respectively). The SM loading can increase methane percentage, but it was not proportional to the loading of SM. 

Similar to the biogas production, average methane production of the control digesters (1849 mg L^−1^) was comparable to 2-1 (1812 mg L^−1^) (not significantly different), but higher (*p* < 0.01) than 1-1 (1700 mg L^−1^) and 1-2 (1542 mg L^−1^), respectively.

#### 3.2.2. Variation of pH, Total Alkalinity and Ammonia

**Figure 3:**
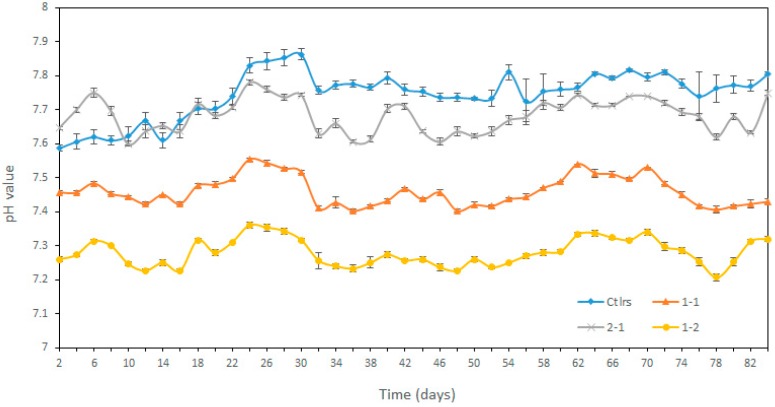
The pH value in different treatments in Test 2.

The control group pH value was lower than 2-1 group before the first HRT, nevertheless, higher after the first HRT (Figure 3). The pH value of 2-1 (7.68 ± 0.05) was the highest when compared with 1-1 (7.46 ± 0.04) and 1-1 (7.28 ± 0.04) in the treatment groups, which was the same as Test 1. A summary of the important variables is presented in Table 3. The VS feeding and VS feedstock of all treatments were 3 g L^−1^ day^−1^ and 63 g L^−1^, respectively. Each of the control and treatments had three replicates. The VS reduction rates of 1-1 (58.36%) and 2-1 (57.46%) were higher than the control (54.81%), except for the 1-2 (50.11%), which can be seen by comparing the VS content of the digestate. 

Similar to Test 1, TA of the control digester had the highest value, 9500mg-CaCO_3_ L^−1^, compared with the treatments, which were all below 7667 mg-CaCO_3_ L^−1^. More SM loading leads to higher TA concentration, suggesting that the CS loading can decrease TA and stabilize the AD process, a benefit of co-digestion. Ammonia concentration of 2-1 (2667 mg L^−1^), 1-1 (1467 mg L^−1^) and 1-2 (867 mg L^−1^) were all lower than the control of single feedstock of SM. More CS addition also could increase stability of the AD process, by decreasing ammonia concentration.

#### 3.2.3. Variation of Carbon, Nitrogen, C/N Ratio, Cellulose and Lignin Degradation Rates

The C/N ratio is an important indicator for controlling the biological treatment system of AD. In Table 4, the control group feedstock contained the highest TC (4.09%) and TN (0.38%) values, but lowest C/N ratio (10.6/1). As expected, the 1-2 feedstock contained the lowest TC (3.41%) and TN (0.15%) contents, but highest C/N ratio (22.7/1). The cellulose content of 1-2 was 42.36 w/w %, 1-1 was 40.39 w/w%, and 2-1 was 38.35 w/w % (Table 4). The lignin contents of different groups were identical. Degradation rates of cellulose were 48.42%, 38.08%, and 34.23% for the 2-1, 1-1, and 1-2 treatment groups, respectively. Degradation rate of lignin of 2-1 was 33.00%, significantly higher (*p* < 0.01) than 1-2 (2.83%), and 1-1 (2.55%). 

#### 3.2.4. Estimation of Carbon Conversion Efficiency

The effective carbon conversion efficiency (ECCE) was calculated using Equation (1):

ECCE = Carbon yield of CH_4_/Total carbon of raw materials and digestate × 100% (1)

Carbon supplied was the carbon content including digestate and raw materials. Methane density is 0.717 g/L (1 atm). The carbon conversion efficiency was of 2-1 was the highest, 40.59%, 1-1 was 39.57%, 1-2 was 37.73% and controls group was seen to have 36.78% (Table 5). Controls group get the highest methane production, but the lowest carbon conversion efficiency. 

### 3.3. Microbial Community Analysis

#### 3.3.1. Microbial Community at Phylum Level

**Figure 4:**
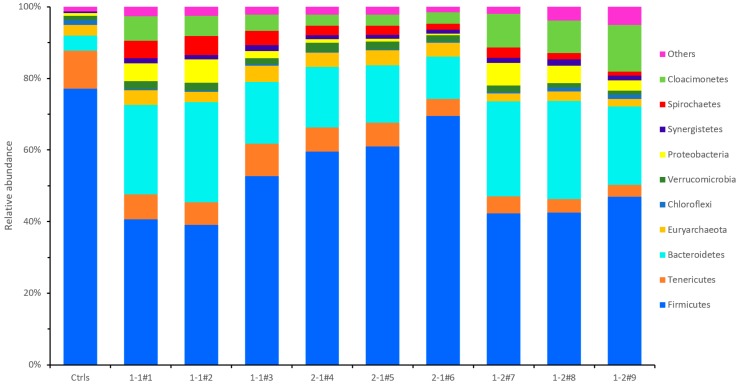
Relative abundance of main taxa at phylum level.

The predominant microorganism community at phylum level (abundance >1%) is depicted with stacked bars in Figure 4. Firmicutes (77.16%), Tenericutes (10.53%), Bacteroidetes (4.23%) and Euryarchaeota (3.00%) were the dominant phyla in the control sample. However, the treatment groups 1-1, 2-1 and 1-2 had more diverse community, and they included Verrucomicrobia, Proteobacteria, Spirochaetae, and Cloacimonetes in addition to those mentioned above. 

Euryarchaeota was found to be the only primary archaea in each sample, which was highly diverse and included methanogens, and the relative abundances of Control, 1-1, 2-1 and 1-2 were 3.00%, 3.89 ± 0.68%, 4.05 ± 0.15%, and 2.36 ± 0.24%, respectively. The relative abundance of Euryarchaeota was significantly higher in group 2-1 than in group 1-2 (*p* < 0.05), which was also likely the reason that the biogas production rate of 2-1 (2414.8 mL d^−1^) was higher than others and very close to the control group (2415 mL d^−1^). Firmicutes was the most abundant in all treatments, averaging 77.16%, 44.13 ± 6.08%, 63.33 ± 4.42%, and 43.95 ± 2.14% for the control, 1-1, 2-1, and 1-2 groups, respectively. The relative abundance of Firmicutes was significantly higher in group 2-1 than in groups 1-1 and 1-2. Proteobacteria was less abundant in the control (0.77%) and 2-1 (0.74 ± 0.23%) groups, but relative abundant in the 1-1 (4.46 ± 1.87%) and 1-2 (4.68 ± 1.39%) groups. The abundances of Synergistetes were 0.40%, 1.50 ± 0.14%, 1.06 ± 0.03% and 1.52 ± 0.20% for the control, 1-1, 2-1, and 1-2 groups, respectively. The phylum Spirochaetes and Cloacimonetes had interesting distribution, they were 0.009% and 0.003% in the control sample, respectively, but Spirochaetes was much abundant in the treatment groups of 1-1 (4.71 ± 0.52%), 2-1 (2.30 ± 0.45%) and 1-2 (1.89 ± 0.72%). The relative abundance of Spirochaetes was significantly higher in group 1-1 than in groups 2-1 (*p* < 0.01) and 1-2 (*p* < 0.05). The abundances of Cloacimonetes were 5.65 ± 0.96%, 3.13 ± 0.04% and 10.5 ± 1.80% for the 1-1, 2-1, and 1-2 groups, respectively. The relative abundance of Cloacimonetes was significantly higher in group 1-2 than in groups 1-1 (*p* < 0.05) and 2-1 (*p* < 0.05). Meanwhile, group 1-1 was significantly higher than 2-1 (*p* < 0.05).

#### 3.3.2. Microbial Community at Genus Level

**Figure 5:**
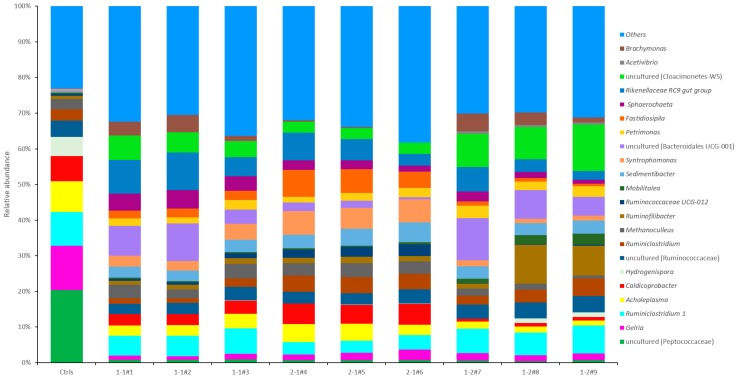
Relative abundance of main taxa at genus level. Uncultured genus microbial was showed family level within bracket because of considerable abundance and importance degree.

The predominant microorganism at genus level (abundance > 1%) is depicted with stacked bar charts in Figure 5. A total of 22 genera are mainly distributed in phylum Firmicutes (13), Bacteroidetes (4), Tenericutes (1), Euryarchaeota (1), Spirochaetae (1), Cloacimonetes (1) and Proteobacteria (1).

Relative high abundance of uncultured genera was found, some were part of family Peptococcaceae. The abundances of Peptococcaceae were 20.38%, 0.80 ± 0.04%, 0.66 ± 0.04% and 0.53 ± 0.10% for the Control, 1-1, 2-1, and 1-2 groups, respectively. The abundances of *Gelria* were 12.40%, 1.27 ± 0.27%, 2.20 ± 0.58% and 1.93 ± 0.19% for the Control, 1-1, 2-1, and 1-2 groups, respectively. The abundances of *Ruminiclostridium 1* were 9.49%, 6.18 ± 0.71%, 3.65 ± 0.30% and 6.98 ± 0.61% for the Control, 1-1, 2-1, and 1-2 groups, respectively. The relative abundance of *Ruminiclostridium 1* was significantly higher in group 2-1 than in groups 1-2 (*p* < 0.01) and 1-1 (*p* < 0.01). The abundance of *Caldicoprobacter* were 7.09%, 3.29 ± 0.24%, 5.60 ± 0.26% and 0.94 ± 0.14% for the Control, 1-1, 2-1, and 1-2 groups, respectively. The 2-1 group was significantly higher than 1-1 group (*p* < 0.01), and 1-1 group was significantly higher than 1-2 group (*p* < 0.01). Some other genera abundances in Control were less than 1%, nevertheless higher values were observed in the treatment groups. The abundances of 1-2 were 6.90 ± 4.03%, 2.31 ± 0.77%, 8.37 ± 2.65% and 10.50 ± 1.80% for the *Ruminofilibacter*, *Mobilitalea*, uncultured (family Bacteroidales UCG-001) and uncultured (family Cloacimonetes-W5), respectively, all were significantly higher than 1-1 (*p* < 0.05) and 2-1 (*p* < 0.05) groups. The abundances of archaea *Methanoculleus* were 2.84%, 3.44 ± 0.67%, 3.65 ± 0.16% and 1.49 ± 0.48% for the Control, 1-1, 2-1, and 1-2 groups, respectively. Groups 2-1 (*p* < 0.01) and 1-1 (*p* < 0.05) were significantly higher than 1-2.

## 4. Discussion

### 4.1. Biogas and Methane Production

Fujita et al. reported CS enhanced gas productivity by more than 65% in mesophilic (39 °C) digestion in comparison with dried SM only, under CSTR, but only adding 2% w/v CS, rather than the feedstock based on VS loading rate (3 g-VS L^−1^ d^−1^) like in this study [9]. The specific gas production is higher than (0.33 ± 0.03 m^3^ kg^−1^ VS_feed_^−1^) that was reported by Cuetos, which is probably because of the different HRT and SM loading rates [11]. As in Test 1, that the methane produced in Test 2 was similar to the biogas production rate, again indicating that the composition of the feedstock did not affect the methane content. The average methane percentages of treatments were in agreement with the 71.5 ± 1.2% value which was reported by Cuetos [11].

### 4.2. Total Alkalinity and Ammonia Concentration

In Test 1, too much of the TA could also influence biogas production, which could be one of the reasons that the C3 biogas production was not directly proportional to the VS loading, when compared with C1 and C2. An advantage of adding CS is that it would decrease TA in the AD process, thus helping to increase the stability of the co-digestion. Besides improving the stability of feedstock, co-digestion can offer the following benefits: dilution of toxic substances, nutrient balance, synergistic effects on microorganisms, increased load of biodegradable organic matter, and producing higher methane yield per unit of digester volume [21]. Among all, TA of 4-2-1#8 was the highest in treatments, which was 11,500 mg CaCO_3_ L^−1^. Ammonia concentration of C3 was 4000 mg NH_3_ L^−1^, indicating that there might have been ammonia inhibition that limited the biogas production. Koster and Lettinga found that when ammonia concentrations were increased in the range of 4051–5734 mg NH_3_ L^−1^, acidogenic populations in the granular sludge was hardly affected while the methanogenic population lost 56.5% of its activity [22]. However, there was not enough replication to validate the findings. In Test 2, high alkalinity concentrations of up to 6500 mg L^−1^ and pH values as high as 7.4, was suggested to have hindered bacterial activity due to toxic effect of the alkalinity [23]. In general, the more CS contents resulted in lower pH value. Ammonia in AD originates mainly from degradation of proteins, urea and nucleic acids, excess ammonia may inhibit aceticlastic and methanogenesis under mesophilic (37 ℃) and thermophilic (55 ℃) [24]. However, it was also documented that the bacteria could be acclimated to ammonium, if it was fed at slowly increasing concentrations [25]. Because the Test 2 was continued from Test 1, which might adapt to higher ammonia concentration. The ammonia concentration of controls was 4700 mg L^−1^, higher than the reported 3075 mg L^−1^ of a test co-digested sewage sludge and piggery manure [26].

### 4.3. C/N Ratio, Cellulose and Lignin Degradation Rate

An early study conducted by Sievers and Brune revealed that the optimal C/N range (adjusted by adding either urea or glucose to the flask digesters) for SM digestion in terms of maximum methane production, was from 15.5/1 to 19/1 [27]. Wu et al. found C/N ratios of 20/1 was optimal for anaerobic co-digesting SM and CS, which could produce maximum biogas and methane production [10]. The optimal C/N ratio of around 14 was found in this experiment, based on biogas production, and again was affected by the digestibility of the cellulose and lignin contents. Corn stover, like most of the lignocellulosic biomasses, is mainly composed of cellulose, hemicellulose and lignin [28]. In Test 2, higher content of CS resulted in higher cellulose content while the lignin contents of different groups were identical. Degradation rates of cellulose were 48.42%, 38.08%, and 34.23% for the 2-1, 1-1, and 1-2 treatment groups, respectively. Degradation rate of lignin of 2-1 was 33.00%, significantly higher (*p* < 0.01) than 1-2 (2.83%), and 1-1 (2.55%). This was likely the main reason that the 2-1 group produced the most biogas and methane. Cheng et al. found the carbon conversion efficiency (including CO_2_) of food waste and sewage sludge was 63.3%. The efficiency may be due to substrate that is more readably degradable (e.g., food wastes and sewage sludge) than SM and CS [29].

### 4.4. Microbial Community at Phylum Level

Amplicon sequencing results indicated Firmicutes was the most dominant phylum. The dominance of Firmicutes in biogas reactors is in accordance with previous studies [30,31]. Firmicutes include syntrophic bacteria that can degrade volatile fatty acids (butyrate and its analogs) [32]. This degradation produces H_2_, which is then utilized by hydrogenotrophic methanogens [33]. Firmicutes also can be attributed to their capability in polysaccharide and oligosaccharide degradation [34]. All above mentioned characteristics can increase biogas production. The abundances of Tenericutes were 10.53%, 7.46 ± 1.20%, 6.07 ± 0.92%, and 3.91 ± 0.64% for the controls, 1-1, 2-1, and 1-2 groups, respectively. Treatment group 1-1 was significantly higher than group 1-2 (*p* < 0.05), while there was no significantly difference among other treatments. Tenericutes comprises a single class, Mollicutes, which were previously classified within the Firmicutes [35]. Recent research reconstructed their metabolism and revealed that, like members of the Mollicutes, these representatives lack a tricarboxylic acid cycle and instead are using anaerobic fermentation of simple sugars for substrate level phosphorylation [36]. Bacteroidetes first intervene in the degradation of proteins and are able to ferment amino acids to acetate [33]. Acetate is then utilized to produce methane by methanogens. The abundances of Bacteroidetes were 4.23%, 23.37 ± 4.54%, 14.91 ± 2.23% and 25.28 ± 2.39% for the control, 1-1, 2-1, and 1-2 groups, respectively. The correlation analysis confirmed that abundance of Bacteroidetes was significantly correlated (*p* < 0.05) with pH values for the treatment groups of 1-1, 2-1 and 1-2. Proteobacteria was relative abundant in the 1-1 (4.46 ± 1.87%) and 1-2 (4.68 ± 1.39%) groups. Proteobacteria was also the predominant phyla detected by Lee et al. in full-scale anaerobic digesters [37]. Spirochaetes was much abundant in the treatment groups of 1-1, 2-1 and 1-2. Lee et al. utilized acetate as substrate in a batch AD experiment. Acetate most stimulated the activity of Spirochaetes, thus, suggesting possible acetate oxidation by a syntrophic acetate oxidation process [38]. There is very little information about the phylum Cloacimonetes. In general, results indicate that higher CS ratio caused more abundant Cloacimonetes, thus a correlation between CS addition and phylum Cloacimonetes can be expected. However, the abundance of phylum Cloacimonetes was not benefiting the biogas production in anaerobic co-digesting of SM and CS in this experiment.

### 4.5. Microbial Community at Genus Level

At genus level, an assumption can be made that adding CS can decrease the abundance of Peptococcaceae. Peptococcaceae is proposed as a new family in the order Eubacteriales to include three genera (*Peptococcus*, *Peptostreptococcus*, and *Ruminococcus*) of presently known gram-positive, anaerobic, coccal organisms, and they were capable of fermenting protein decomposition products [39]. The abundances of *Gelria* were 12.40%, 1.27 ± 0.27%, 2.20 ± 0.58% and 1.93 ± 0.19% for the Control, 1-1, 2-1, and 1-2 groups, respectively. *Gelria* can degrade glutamate to ammonia [40]. The control group has the highest abundance, which indicates a significant correlation with ammonia content. *Ruminiclostridium* genus contains anaerobic bacteria, which is believed to develop different strategies to depolymerize the cellulose and the related plant cell wall polysaccharides [41]. The abundances of *Ruminiclostridium* *1* were 9.49% for the Control group. The results indicated that addition of CS could also increase *Ruminiclostridium 1* abundance in the treatment groups, which is consistent with the function that Ravachol et al. reported. More CS loading leads to greater *Acholeplasma* abundance [41]. *Acholeplasma* is also implicated in the first step of degrading polycyclic aromatic hydrocarbons [42]. *Caldicoprobacter* was isolated from an Algerian hot spring that is an obligatory heterotroph fermenting sugars. End-products from glucose fermentation were acetate, lactate, ethanol, CO_2_, and H_2_ [43]. *Caldicoprobacter* was also detected from continuous stirred tank reactors digesting chicken manure at mesophilic temperature [44]. *Ruminofilibacter*, *Mobilitalea*, uncultured (family Bacteroidales UCG-001) and uncultured (family Cloacimonetes-W5) for treatment groups were extreme significant (*p* < 0.01) higher than control groups. Group 1-2 was seen to have the highest abundance in all treatments. *Ruminofilibacter* is hydrolytic bacteria while some species process hemicellulolytic activities [30]. *Mobilitalea* belongs to family Lachnospiraceae. Lachnospiraceae is VFA-producing bacteria, one of many unclassified species that belonged to a group comprising soluble polysaccharide-degrading bacteria [45]. 

### 4.6. Outlook of Full-Scale Anaerobic Co-Digestion SM with CS

In full-scale anaerobic digestion studies, domestic refuse, municipal solid waste (MSW) and cow manure and cattle manure with agricultural residues were systematically [46,47,48]. In this paper, anaerobic co-digestion with swine manure and corn stover was studied, and optimal loading rate and SM/CS ratio were confirmed, which was beneficial for future research in full-scale AD. Because of lower density of CS, it will float to the top of reactor around 4 h in a preliminary test. Thus, the semi-continuous stirred was necessary, the stirring frequency and energy balance should be further studied in subsequent full-scale research. 

## 5. Conclusions

An extended stabilization of anaerobic co-digestion with SM and CS substrates was conducted. The optimal VS loading, SM/CS and C/N were 3 g-VS L^−1^ d^−1^, 2/1 and 14/1, respectively. The highest effective carbon efficiency was also detected at SM/CS = 2/1. High biogas production wasn’t representative of effective energy and carbon conversion efficiency. Microbial diversity increased with CS loading. Amino-acid-degrading bacteria was abundant in the treatment group. Archaea *Methanoculleus* was the most abundant microorganism beneficial for producing biogas. On the contrary, hydrolytic and acid-producing bacteria (*Ruminofilibacter*, *Mobilitalea*, *Sedimentibacter*, *Sphaerochaeta*) were observed to be associated with lower biogas production. 

## Figures and Tables

**Table 1:** Characteristic of the feedstocks, swine manure (SM) and corn stover (CS).

	pH	Moisture (%)	TS (%) ^c^	VS (%) ^d^	TC (%) ^e^	TN (%) ^f^	Cellulose (w/w%)	lignin (w/w%)
SM ^a^	5.33 ± 0.23	69.87 ± 3.27	25.89 ± 0.09	82.0 2 ± 0.18	13.30 ± 1.40	1.25 ± 0.11	8.10	3.31
CS ^b^	-	8.80 ± 0.10	90.95 ± 0.05	95.13 ± 0.25	41.95 ± 0.25	0.49 ± 0.03	46.10	10.97

^a^ swine manure. ^b^ corn stover. ^c^ total solid. ^d^ volatile solid. ^e^ total carbon. ^f^ total nitrogen.

**Table 2:** Summary of biogas and methane yield, and other important variables of Test 1 (7 May–6 July 2017, 72–120 days).

	Units	C 1 ^a^	C 2	C 3	2-1-1#1	2-2-1#2	2-1-2#3	3-1-1#4	3-2-1#5	3-1-2#6	4-1-1#7	4-2-1#8	4-1-2#9
VS digestate	g L^−1^	14.5	28.6	39.9	22.3	20.1	20.5	26.3	27.3	31.2	35.9	39.5	42.4
VS reduction	%	65.5	54.6	52.5	46.9	52.1	51.2	58.3	56.7	50.5	57.3	53.0	49.5
Biogas	mL d^−1^	1856	2658	2804	1559	1640	1223	2258	2400	1983	2878	3112	2612
Biogas	mL g^−1^ VS _des_^b^	97.8	112.0	92.2	114.7	108.5	82.4	89.2	97.4	90.4	86.7	101.3	91.0
CH_4_ content	%	75.6	73.1	70.5	73.6	73.6	74.3	70.3	70.3	69.3	67.9	69.1	67.3
CH_4_	mL d^−1^	1405	1942	1971	1148	1209	909	1576	1689	1368	1952	2147	1749
CH_4_	mL g^−1^ VS _des_	74.0	81.8	64.8	84.4	80.0	61.3	62.2	68.6	62.3	58.8	69.9	61.0
pH	-	7.61	7.87	7.86	7.27	7.38	7.08	7.38	7.57	7.17	7.52	7.72	7.27
TA ^c^	mg L^−1^	10,500	16,000	18,000	6500	7000	5000	7500	9000	6500	9000	11,500	7500
Ammonia	mg L^−1^	1600	2400	4000	800	800	800	800	800	800	800	1600	800
^a^ control groups. ^b^ VS-destroyed. ^c^ total alkalinity.													

**Table 3:** Summary of important variables was presented in Test 2 (3 g-VS L^−1^ day^−1^).

	Units	Ctrls ^a^	1-1	2-1	1-2
VS digestate	g/L	28.47 ± 0.82	26.23 ± 0.12	26.80 ± 0.36	31.43 ± 0.47
VS reduction	%	54.81 ± 1.30	58.36 ± 0.20	57.46 ± 0.56	50.11 ± 0.75
Biogas	mL d^−1^	2415.2 ± 37.42	2284.4 ± 23.62	2414.8 ± 83.38	2166.0 ± 181.62
Biogas	mL g^−1^ VS _des_	101.41 ± 2.71	90.04 ± 0.70	96.72 ± 4.32	99.51 ± 8.85
CH_4_ content	%	76.56 ± 1.35	74.41 ± 1.40	75.02 ± 1.17	71.25 ± 1.25
CH_4_	mL d^−1^	1849.0 ± 31.08	1699.8 ± 11.21	1811.6 ± 66.99	1542.3 ± 118.53
CH_4_	mL g^−1^ VS _des_	77.63 ± 1.85	67.00 ± 0.33	72.56 ± 3.40	70.84 ± 5.73
pH	-	7.75 ± 0.07	7.46 ± 0.04	7.68 ± 0.05	7.28 ± 0.04
TA**^b^**	mg L^−1^	9500 ± 354	7333 ± 118	7667 ± 236	6083 ± 118
Ammonia	mg L^−1^	4700 ± 141	1467 ± 340	2667 ± 499	867 ± 94

^a^ control groups. ^b^ total alkalinity.

**Table 4:** Summary of TC, TN, C/N, cellulose and lignin degradation rate in Test 2.

	Raw materials			Cellulose (w/w%)		Lignin (w/w%)	
	TC (%)	TN (%)	C/N	Substrate	Digestate	Substrate	Digestate
Ctrls	4.09	0.38	10.64	34.26	-	13.11	-
1-1	3.59	0.21	17.10	40.39	25.01	12.54	12.22
2-1	3.76	0.27	13.93	38.35	19.78	13.03	8.73
1-2	3.41	0.15	22.73	42.36	27.86	12.73	12.37

**Table 5:** The carbon conversion efficiency in Test 2 (per day ^a^).

	Raw materials			TC of SM (g)	TC of CS (g)	TC of Digestate (%)	TC of Digestate (g)	TC of CH_4_ (g)	ECCE (%) ^b^
	SM (g)	CS (g)	Distilled water (g)						
Ctrls**^c^**	19.42	-	46.08	2.58	-	0.63	8.25	1.02	36.78
1-1	9.70	2.40	53.40	1.29	1.01	0.49	6.42	0.91	39.57
2-1	12.95	1.60	50.95	1.72	0.67	1.29	16.89	0.97	40.59
1-2	6.48	3.20	55.82	0.86	1.34	0.54	7.07	0.83	37.73

^a^ all data was calculated on a daily basis. ^b^ ECCE: effective carbon conversion efficiency. ^c^ control groups.

## References

[1] Chen Y., Cheng J.J., Creamer K.S. (2008). Inhibition of anaerobic digestion processes: A review. Biores. Tech..

[2] Riaño B., Garcíagonzález M.C. (2014). On-farm treatment of swine manure based on solid-liquid separation and biological nitrification-denitrification of the liquid fraction. J. Environ. Manag..

[3] Lyberatos G., Skiadas I.V. (1999). Modelling of anaerobic digestion-a review. Glob. Nest. Int. J..

[4] Wang M., Zhang X.Y., Zhou J., Yuan Y.X., Dai Y.M., Li D., Liu X.F., Yan Z.Y. (2017). The dynamic changes and interactional networks of prokaryotic community between co-digestion and mono-digestions of corn stalk and pig manure. Biores. Tech..

[5] Mata-Alvarez J., Dosta J., Romero-Güiza M.S., Fonoll X., Peces M., Astals S. (2014). A critical review on anaerobic co-digestion achievements between 2010 and 2013. Renew. Sustain. Energy Rev..

[6] FAOSTAT. http://faostat3.fao.org/.

[7] Sekhon R.S., Breitzman M.W., Silva R.R., Santoro N., Rooney W.L., De-Leon N., Kaeppler S.M. (2016). Stover composition in maize and sorghum reveals remarkable genetic variation and plasticity for carbohydrate accumulation. Front. Plant Sci..

[8] Spyridon M., Keri B.C., Francisco J.A., Kipling S.B., Jeff M.N., James R.F., Douglas L.K. (2016). Carbohydrate and nutrient composition of corn stover from three southeastern USA locations. Biomass Bioenergy.

[9] Fujita M., Scharer J.M., Mooyoung M. (1980). Effect of corn stover addition on the anaerobic digestion of swine manure. Agric. Wastes.

[10] Wu X., Yao W.Y., Zhu J., Miller C. (2010). Biogas and CH_4_ productivity by co-digesting swine manure with three crop residues as an external carbon source. Biores. Tech..

[11] Cuetos M.J., Fernández C., Gómez X., Morán A. (2011). Anaerobic co-digestion of swine manure with energy crop residues. Biotech. Bioprocess Eng..

[12] Zhang T., Mao C.L., Zhai N., Wang X., Yang G. (2015). Influence of initial pH on thermophilic anaerobic co-digestion of swine manure and maize stalk. Waste Manag..

[13] Gao Y.M., Yang A.Y., Bao J., Ma R.X., Yan L., Wang Y.J., Wang W.D. (2017). Bioreactor performance and microbial community dynamics in a production-scale biogas plant in Northeast of China. Int. J. Agri. Biol. Eng..

[14] EPA (2001). Total, fixed, and Volatile Solids in Water, Solids, and Biosolids.

[15] AOAC (1997). AOAC Official Method 973.18, Fiber (Acid Detergent) and Lignin in Animal Feed.

[16] Nogueira R.G.S., Teng T.L., Wang H.Q., Rodrigues P.H.M. (2019). Performance, microbial community analysis and fertilizer value of anaerobic co-digestion of cattle manure with waste kitchen oil. Appl. Eng. Agric..

[17] Ericsson A.C., Davis D.J., Franklin C.W., Hagan C.E. (2015). Exoelectrogenic capacity of host microbiota predicts lymphocyte recruitment to the gut. Physiol. Genom..

[18] Parada A.E., Needham D.M., Fuhrman J.A. (2016). Every base matters: Assessing small subunit rRNA primers for marine microbiomes with mock communities, time series and global field samples. Environ. Microbiol..

[19] Apprill A., Mcnally S., Parsons R., Weber L. (2015). Minor revision to V4 region SSU rRNA 806R gene primer greatly increases detection of SAR11 bacterioplankton. Aquatic Microbial Ecol..

[20] Agler M.T., Wrenn B.A., Zinder S.H., Angenent L.T. (2011). Waste to bioproduct conversion with undefined mixed cultures: The carboxylate platform. Trends Biotech..

[21] Nkemka V.N., Murto M. (2010). Evaluation of biogas production from seaweed in batch tests and in UASB reactors combined with the removal of heavy metals. J. Environ. Manag..

[22] Koster I.W., Lettinga G. (1988). Anaerobic digestion at extreme ammonia concentrations. Biol. Wastes.

[23] Albertson O.E. (1961). Ammonia nitrogen and the anaerobic environment. J. Water Pollut. Control. Fed..

[24] Hailin T., Loannis A.F., Konstantinos K., Irini A. (2017). Effect of different ammonia sources on aceticlastic and hydrogenotrophic methanogens. Biores. Tech..

[25] Melbinger N.R., Donnellon J., Zablatzky H.R. (1971). Toxic effects of ammonia nitrogen in high-rate digestion [with Discussion]. J. Water Pollut. Control. Fed..

[26] Velsen A.F.M.V. (1979). Adaptation of methanogenic sludge to high ammonia-nitrogen concentrations. Water Res..

[27] Sievers D.M., Brune D.E. (1978). Carbon/nitrogen ratio and anaerobic digestion of swine waste. Trans. ASAE.

[28] Bondesson P.M., Galbe M., Zacchi G. (2013). Ethanol and biogas production after steam pretreatment of corn stover with or without the addition of sulphuric acid. Biotech. Biofuels.

[29] Cheng J., Ding L., Lin R., Yue L., Liu J.Z., Zou J.H., Cen K. (2016). Fermentative biohydrogen and biomethane co-production from mixture of food waste and sewage sludge: Effects of physiochemical properties and mix ratios on fermentation performance. Appl. Energy.

[30] Kröber M., Bekel T., Diaz N.N., Goesmann A., Jaenicke S., Krause L. (2009). Phylogenetic characterization of a biogas plant microbial community integrating clone library 16S-rDNA sequences and metagenome sequence data obtained by 454-pyrosequencing. J. Biotechnol..

[31] Schlüter A., Bekel T., Diaz N.N., Dondrup M., Eichenlaub R., Gartemann K.H., Goesmann A. (2008). The metagenome of a biogas-producing microbial community of a production-scale biogas plant fermenter analysed by the 454-pyrosequencing technology. J. Biotech..

[32] Vartoukian S.R., Palmer R.M., Wade W.G. (2007). The division “Synergistes”. Anaerobe.

[33] Rivière D. (2009). Towards the definition of a core of microorganisms involved in anaerobic digestion of sludge. ISME J..

[34] Krause L., Diaz N.N., Edwards R.A., Gartemann K.H., Krömeke H., Neuweger H. (2008). Taxonomic composition and gene content of a methane-producing microbial community isolated from a biogas reactor. J. Biotech..

[35] Garrity G.M., Bell J.A., Lilburn T., Brenner D.J., Krieg N.R., Staley J.T., Garrity G.M. (2005). The revised road map to the manual. Bergey’s Manual^®^ of Systematic Bacteriology.

[36] Skennerton C.T., Haroon M.F., Briegel A., Shi J., Jensen G.J., Tyson G.W., Orphan V.J. (2016). Phylogenomic analysis of Candidatus ‘*Izimaplasma*’ species: Free-living representatives from a Tenericutes clade found in methane seeps. ISME J..

[37] Lee S.H., Kang H.J., Lee Y.H., Lee T.J., Han K., Choi Y. (2012). Monitoring bacterial community structure and variability in time scale in full-scale anaerobic digesters. J. Environ. Monit..

[38] Lee S.H., Park J.H., Kang H.J., Lee Y.H., Lee T.J., Park H.D. (2013). Distribution and abundance of *Spirochaetes* in full-scale anaerobic digesters. Biores. Tech..

[39] Rogosa M. (1971). Peptococcaceae, a new family to include the gram-positive, anaerobic cocci of the genera *Peptococcus*, *Peptostreptococcus*, and *Ruminococcus*. Int. J. Syst. Bacteriol..

[40] He Q., Li L., Zhao X.F., Li Q., Wu D., Peng X.Y. (2017). Investigation of foaming causes in three mesophilic food waste digesters: Reactor performance and microbial analysis. Sci. Rep..

[41] Ravachol J., Borne R., Meynialsalles I., Soucaille P., Pagès S., Tardif C. (2015). Combining free and aggregated cellulolytic systems in the cellulosome-producing bacterium Ruminiclostridium cellulolyticum. Biotech. Biofuels.

[42] Braun F., Hamelin J., Bonnafous A., Delgenès N., Steyer J.P., Patureau D. (2015). Similar PAH fate in anaerobic digesters inoculated with three microbial communities accumulating either volatile fatty acids or methane. PLoS ONE.

[43] Bouanane-Darenfed A., Fardeau M.L., Grégoire P., Joseph M., Kebbouchegana S., Benayad T. (2011). *Caldicoprobacter algeriensis* sp. nov. a new thermophilic anaerobic, xylanolytic bacterium isolated from an algerian hot spring. Curr. Microbiol..

[44] Ziganshina E.E., Ibragimov E.M., Vankov P.Y., Miluykov V.A., Ziganshin A.M. (2016). Comparison of anaerobic digestion strategies of nitrogen-rich substrates: Performance of anaerobic reactors and microbial community diversity. Waste Manag..

[45] Nyonyo T., Shinkai T., Mitsumori M. (2014). Improved culturability of cellulolytic rumen bacteria and phylogenetic diversity of culturable cellulolytic and xylanolytic bacteria newly isolated from the bovine rumen. FEMS Microbiol. Ecol..

[46] Gregor D.Z., Natasa U.Z., Milenko R. (2008). Full-scale anaerobic digestion of organic waste and municipal sludge. Biomass Bioenergy.

[47] Alessandro C., Francesco D.B., Sonia L. (2018). Dry anaerobic digestion of cow manure and agricultural products in a full-scale plant: Efficiency and comparison with wet fermentation. Waste Manag..

[48] Cavinato C., Fatone F., Bolzonella D., Pavan P. (2010). Thermophilic anaerobic co-digestion of cattle manure with agro-wastes and energy crops: Comparison of pilot and full scale experiences. Biores. Tech..

